# The long-term effects of anti-vascular endothelial growth factor therapy on the optical coherence tomography angiographic appearance of neovascularization in age-related macular degeneration

**DOI:** 10.1186/s40942-020-00242-z

**Published:** 2020-08-20

**Authors:** Emily S. Levine, Eugenia Custo Greig, Luísa S. M. Mendonça, Shilpa Gulati, Ivana N. Despotovic, A. Yasin Alibhai, Eric Moult, Nora Muakkassa, Maddalena Quaranta-El Maftouhi, Adil El Maftouhi, Usha Chakravarthy, James G. Fujimoto, Caroline R. Baumal, Andre J. Witkin, Jay S. Duker, M. Elizabeth Hartnett, Nadia K. Waheed

**Affiliations:** 1 New England Eye Center, Tufts Medical Center, Boston, MA USA; 2 Tufts University School of Medicine, Boston, MA USA; 3 Yale School of Medicine, New Haven, CT USA; 4 Department of Ophthalmology, Federal University of São Paulo, São Paulo, Brazil; 5 Department of Electrical Engineering and Computer Science, Research Laboratory of Electronics, Massachusetts Institute of Technology, Cambridge, MA USA; 6 Denver Eye Surgeons, Lakewood, CO USA; 7 Centre Ophtalmologique Rabelais, Lyon, France; 8 Centre for Public Health, Queen’s University Belfast, Belfast, UK; 9 John A. Moran Eye Center, University of Utah, Salt Lake City, UT USA; 10 Department of Ophthalmology, Tufts Medical Center, 800 Washington Street, Box 450, Boston, MA 02111 USA

**Keywords:** Macular neovascularization, Optical coherence tomography angiography, Anti-VEGF

## Abstract

**Background:**

The short-term effects of anti-vascular endothelial growth factor (anti-VEGF) treatment on macular neovascularization (MNV) morphology is well described, but long-term studies on morphologic changes and correlation of such changes to the type of MNV have not been conducted. This study aims to determine if different types of MNVs in neovascular AMD (nAMD) behave differently with anti-VEGF treatment as visualized on optical coherence tomography angiography (OCTA).

**Methods:**

Treatment-naïve nAMD patients were retrospectively screened for baseline and follow-up OCTA imaging 10 or more months after initial treatment. Images were graded for MNV type, area, activity, mature versus immature vessels, vessel density, presence of atrophy, atrophy location and area. Growth rate was calculated as the percent change in lesion area from baseline over the years of follow-up. In addition, the occurrence of complete regression and the percent of lesions that grew, remained stable, and shrunk per type was also evaluated.

**Results:**

Forty-three eyes from 43 patients with a mean follow-up of 2 years were evaluated. On structural OCT, 26 lesions were classified as pure type 1 MNVs, 12 MNVs had a type 2 component, and 5 MNVs had a type 3 component. Of these cases, 2 mixed-type MNVs were considered to have completely regressed. There was no significant differences in MNV area and growth rate between type 1 and type 2 lesions, but all cases of type 3 lesions shrunk in the follow-up period. There was no correlation between the number of injections per year and growth rate, endpoint MNV area or endpoint activity status for any MNV type. There was no significant association between the development of atrophy and the number of injections, baseline MNV area, baseline vessel density, or lesion growth rate.

**Conclusions:**

In nAMD, complete regression of an MNV network exposed to anti-VEGF is rare. This work emphasizes the role of anti-VEGF as anti-leakage rather than vascular regression agents in nAMD.

## Background

Age-related macular degeneration (AMD) is a major cause of blindness in older adults. Neovascular AMD (nAMD) is an advanced stage of the disease that can lead to vision loss due to macular damage from abnormal blood vessel exudation. There are three main forms of neovascularization that can occur in nAMD based on where the new blood vessels originate and grow [1]. Type 1 and type 2 neovascularizations originate from the choroidal vasculature and are located under the retinal pigment epithelium (RPE) and in the subretinal space, respectively. Type 3 neovascularization is thought to originate from the deep retinal capillary plexus, grow downward into the neurosensory retina, and may form retinal-choroidal anastomoses at later stages [2–4]. Many neovascular lesions in AMD are a mixture of subtypes and are dubbed mixed neovascularization. For example, type 2 lesions often occur in the context of type 1 vessels that have migrated across the RPE into the subretinal space [1–3]. Mixed and pure type neovascularizations have been grouped together under the umbrella term macular neovascularization (MNV) [5].

Anti-vascular endothelial growth factor (anti-VEGF) therapy is currently the mainstay treatment for a wide range of retinal pathologies, including MNVs associated with nAMD. Anti-VEGF treatment is considered to have two main beneficial effects on retinal pathologies: reduction in vascular permeability and decreased neovascular growth [6]. Anti-permeability effects of anti-VEGF can easily be detected by measuring fluid on optical coherence tomography (OCT) or leakage on fluorescein angiography (FA), and reduction in vascular permeability has uniformly been used as a surrogate for anti-VEGF efficacy in retinal diseases. However, the effects of anti-VEGF treatment on abnormal vasculature can vary from disease to disease. For example, MNV lesions secondary to some disease states such as pathologic myopia regress rapidly with anti-VEGF exposure [7–10]. Similarly, studies looking at the effect of anti-VEGF treatment on the retinal neovascularization seen in proliferative diabetic retinopathy (PDR) show rapid and sustained resolution of retinal neovascularization after anti-VEGF therapy [11, 12].

Conversely, MNVs in nAMD respond more variably to anti-VEGF treatment. Some studies have shown decreased neovascular membrane area, pruning of small capillaries, and expansion of vessel caliber with anti-VEGF treatment for MNVs in nAMD [13–17]. However, using gold-standard FA, the landmark trials MARINA and ANCHOR showed that, on average, anti-VEGF therapy curbed the growth of and reduced the leakage from neovascular lesions but did not stop the expansion of MNVs over time in all cases [18, 19].

OCT angiography (OCTA) offers a non-invasive and depth-resolved alternative to gold-standard dye-based angiography, yielding high-resolution images that can localize vascular complexes to specific retinal and choroidal layers, which allows for the detailed study of MNV morphology [20, 21]. Contemporary studies have applied OCTA to further explore the behavior of MNVs associated with nAMD [13, 22]. OCTA imaging suggests that some type 3 MNVs may completely regress with chronic anti-VEGF treatment [23, 24]. Short-term studies of anti-VEGF effects on type 1 and type 2 MNVs using OCTA report that anti-VEGF can prevent further MNV growth [25]. However, these studies only followed MNVs for a limited period of time and did not address the effects of prolonged anti-VEGF exposure. Studies on type 1 MNVs using OCTA have shown that they do not regress with anti-VEGF therapy [1, 26]. Studies on chronically treated MNVs have shown arteriolization of the vasculature, but such cross-sectional studies suffer from a selection bias [27]. There has not yet been a systematic evaluation of the long-term regression and growth rates across the three different types of MNVs in nAMD exposed to anti-VEGF. The purpose of this study is to investigate the long-term effects of anti-VEGF therapy on MNV growth or regression and to address whether the three lesion types respond differently to anti-VEGF treatment. Based on evidence from pre-clinical studies, we also propose hypotheses to explain the observed clinical outcomes and provide suggestions for future directions.

## Methods

### Subjects

This was a retrospective cohort study of eyes with treatment naïve nAMD that were treated with anti-VEGF injections and imaged at the New England Eye Center, Boston, MA or at the Centre Ophtalmologique Rabelais, Lyon, France between December 2014 and December 2018. Both institutions received institutional review board approval and research was performed in accordance with the Declaration of Helsinki and the Health Insurance Portability and Accountability Act.

Patients were considered for inclusion in this study if they met the following criteria: (1) the patient had a treatment naïve MNV, (2) OCTA imaging was performed at baseline prior to the initiation of anti-VEGF treatment, (3) OCTA imaging was available at least 10 months after the baseline visit, and (4) the patient received any number of anti-VEGF injections over the follow-up period either per a treat and extend, pro re nata, or a monthly injection protocol. Exclusion criteria were (1) evidence of other retinal vascular disorders (e.g. diabetic retinopathy, branch retinal artery occlusion, etc.) as noted in the patient chart and (2) an inability to interpret the baseline or endpoint OCTA image due to significant image artifact or hemorrhage blocking the signal. Patients were not excluded if they had received anti-VEGF injections in the fellow eye. Best corrected visual acuity (BCVA) at the baseline and endpoint visits were converted to logMAR and recorded for each patient. The number of anti-VEGF injections received in the follow-up period, as well as the type of agent given (ranibizumab, aflibercept, bevacizumab), was determined by chart review and recorded. If a patient was noted to have had treatment with more than one anti-VEGF agent, the agent type attribute was assigned as “combination”.

### Image analysis and outcome measurements

Images were acquired on one of three OCTA devices and qualitatively analyzed in each devices’ respective review software. The three devices used in this study were: the spectral domain RTVue XR Avanti with Angiovue (Optovue, Inc., Fremont, CA), the spectral domain Cirrus HD-OCT 5000 (Carl Zeiss Meditec, Dublin, CA), and the swept-source PLEX Elite 9000 (Carl Zeiss Meditec, Dublin, CA). The baseline image was defined as the first OCTA image available for a newly diagnosed MNV prior to anti-VEGF treatment. The endpoint image was selected as the most recent OCTA image on file that met image quality standards. The 6 × 6 mm macular scan pattern was the default for analysis, but the 3 × 3 mm macular scan pattern was used if the 6 × 6 mm image was of poor quality or unavailable.

Images were graded independently by 2 of 3 qualified graders (ESL, SG, ID). Adjudication was performed if graders were in disagreement over a categorical metric or if there was greater than a 10% difference between quantitative measurements. If there was still disagreement between graders, a retina specialist reviewed the case and made a final judgement (LM, NKW).

The following qualitative outcomes were assessed at both the baseline and endpoint visits: the presence of immature or mature vessels, lesion activity status, and the presence and location of macular atrophy (e.g. subfoveal, parafoveal, or macular). Immature vessels were defined as fine, branching vessels (Fig. 6a, green arrow) whereas mature vessels were defined as wider, snake-like vessels (Fig. 4a, green arrow) [17, 28]. A lesion was defined as active if there was subretinal or intraretinal fluid present. If there was no fluid present at baseline, the activity status was deemed to be subclinical. Such patients would not have received treatment at the baseline but would have received treatment in the interim before the endpoint. If there was no fluid present at the endpoint, the activity status was deemed inactive. Additionally, MNV type was determined at baseline. Because MNV morphology alters with natural history and with treatment, the predominant lesion type was only determined in the treatment-naïve baseline images. The structural features used to classify MNV type are as follows: type 1 components were identified as large and/or irregular pigment epithelial detachments; a type 2 component was identified as the presence of dense subretinal material in the subretinal space; a type 3 component was identified as intraretinal hyperreflectivity with associated intraretinal cysts (hyporeflective spaces) [5]. Pixels representing flow needed to overlie such structural findings in order for an MNV to be considered as present. For the purposes of analysis, mixed-type lesions with a type 2 component were grouped in with type 2 MNVs while mixed-type lesions with a type 3 component were grouped in with type 3 MNVs.

Quantitative outcomes were assessed in a custom software called the OCTA Analysis Toolbox (OAT). This software permits tracing over en face OCTA images and custom image thresholding for each device and scan size. The OAT software was used to allow for uniform analysis across the different imaging platforms utilized in this study. The outcomes measured in the OAT software included MNV area, MNV vessel density, and atrophy area; these outcomes are defined below.

To analyze MNV area and vessel density, images were first manually segmented on the OCTA device to obtain the best possible en face view of the neovascularization. En face images were then imported into the OAT software, where the MNV lesion was manually traced. If MNV vessels were not well visualized on the en face image, the tracing was performed while scrolling through the corresponding OCTA B-scans in the device review software where the pixels representing flow could be followed. For such images, MNV vessel density measurements were excluded from analysis. Lesion area was defined as the total area in mm^2^ encompassed by the MNV tracing. Vessel density was defined as the ratio of white pixels (representing flow) to total pixels within MNV tracing after binarization. An optimal global threshold for binarization, which allows separation of vessel pixels from non-vessel pixels, was empirically selected and applied to all images during analysis.

Atrophy was defined as the presence of signal hypertransmission on the B-scan and accompanying RPE attenuation. Because the extent of these two features were not measured, incomplete RPE and outer retinal atrophy (iRORA) and complete RPE and outer retinal atrophy (cRORA) were not distinguished [29]. Atrophy was measured on a custom slab 320 μm below Bruch’s membrane. This allowed for the full extent of hypertransmission to be visualized on the en face OCT image as a region of relative brightness. This structural image was then imported into the OAT software where areas of atrophy were traced. The software calculated the area of atrophy to be the total mm^2^ encompassed within all traced areas.

### Statistical analysis

The growth rate was calculated as the percent change in lesion area (baseline area minus endpoint area divided by the baseline area) divided by the follow-up duration in years. This normalized growth to initial lesion size. Furthermore, lesions were subdivided into three growth categories including those that remained stable (≤ 10% change from baseline to endpoint), shrunk (> 10% reduction from baseline) or grew (> 10% increase from baseline). The percentage of lesions per growth category was calculated for each MNV type.

A Kruskal–Wallis test was employed to compare continuous variables among MNV types, including the number of injections, follow up duration, growth rate, baseline and endpoint MNV areas, baseline and endpoint vessel densities, and baseline and endpoint visual acuities. If the Kruskal–Wallis test revealed a significant difference across the three MNV types for a given variable, a Mann–Whitney U test for pairwise comparison was performed to inform where a significant difference occurred. A Wilcoxon signed rank test was also performed to compare the baseline and endpoint MNV areas and vessel densities within a given MNV type. Spearman’s rank correlation was used to assess the relationship between endpoint MNV area and number of injections, baseline vessel density and growth rate, and growth rate and number of injections. A Chi-square test was used to assess categorical variables per MNV type including vessel maturity at baseline, complete regression at the endpoint, endpoint activity status, and growth category. Logistic regression modeling was performed to assess the correlation between development of endpoint atrophy and various continuous variables including baseline MNV area, baseline vessel density, number of injections, follow-up duration, and growth rate. This logistic regression analysis was performed after excluding patients with baseline atrophy and after controlling for the number of anti-VEGF injections per subject.

Finally, a Kruskal–Wallis test was utilized to assess potential differences across anti-VEGF agent type. Growth rate, endpoint MNV area, and endpoint activity status across agent type (bevacizumab, aflibercept, ranibizumab, or a combination of agents) were assessed in the overall cohort. MNV type was not accounted for in this sub-analysis to avoid further sample size reduction.

A p value of ≤ 0.05 was considered significant. All statistical analyses were performed using RStudio version 1.1.463.

## Results

Of the 966 nAMD patients screened for this study, 52 met inclusion criteria. The most common reason for exclusion was lack of treatment-naïve status at the time of initial OCTA imaging. Forty-three eyes (26 OD, 17 OS) from 43 patients with at least 10 months of follow-up were successfully evaluated (Table 1). Twelve cases were generated by the RTVue XR Avanti (Avanti) at both baseline and endpoint, 16 cases were generated by the Cirrus HD-OCT 5000 (Cirrus) at both baseline and endpoint, and 15 cases used a different device for the baseline and endpoint imaging (Avanti-Cirrus: 11 cases, Cirrus-PLEX Elite 9000 [PLEX]: 2 cases, PLEX-Avanti: 2 cases).

**Table 1:** Demographic characteristics of all subjects combined and stratified by macular neovascularization (MNV) type

	Total (43 eyes)	Type 1 (26 eyes)	Type 2 (12 eyes)	Type 3 (5 eyes)	*p*
Mean age (95% CI), years	78 (76.14–80.46)	77.44 (74.38–80.5)	80 (76.25–83.75)	78.2 (73.59–82.81)	0.529
Sex, male/female	14/28	9/16	4/9	1/4	0.774
Mean follow-up (95% CI), years	2.09 (1.8–2.38)	2.01 (1.67–2.35)	2.49 (1.79–3.19)	1.55 (1.04–2.06)	0.276
Mean anti-VEGF injections (95% CI), n	10.58 (8.07–13.09)	10.31 (7.4–13.22)	10.93 (6.14–18.52)	8 (3.12–12.88)	0.754
Anti-VEGF agent, n					
Aflibercept	18	12	5	1	
Bevacizumab	7	5	1	1	
Ranibizumab	5	1	2	2	
Combination	14	7	6	1	
Mean visual acuity, logMAR					
Baseline	0.691	0.549	1.110	0.426	0.012*
Endpoint	0.733	0.620	1.147	0.329	0.006**

*CI* confidence interval

*p ≤ 0.05, **p ≤ 0.01

**Fig. 1:**
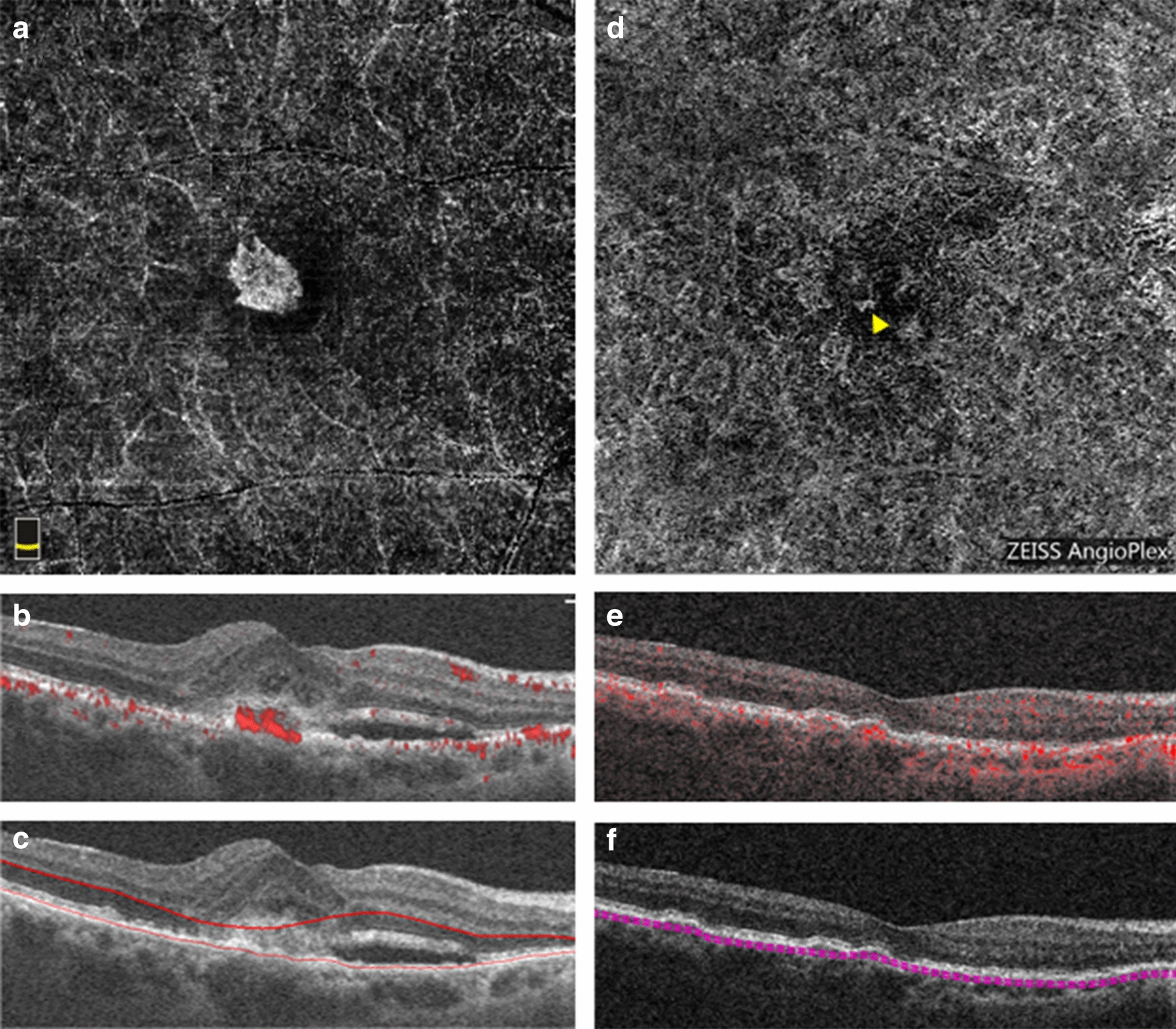
Optical coherence tomography angiography (OCTA) imaging of a pure type 2 MNV. **a**–**c** Baseline OCTA imaging from the RTVue XR Avanti device. **a** En face OCTA image of the outer retinal slab depicting the MNV network. **b** Corresponding B-scan through the lesion revealing subretinal flow signal. **c** Corresponding B-scan through the lesion showing segmentation lines for the outer retinal slab. **d**–**f** Endpoint OCTA imaging from the Cirrus HD-OCT 5000 device 44 months after the baseline imaging and after 20 total intravitreal injections of anti-VEGF. **d** En face OCTA image of the choriocapillaris slab depicting the MNV flow (yellow arrow). **e** Corresponding B-scan through the lesion revealing now sub-RPE flow signal. **f** Corresponding B-scan through the lesion showing segmentation lines for the choriocapillaris

**Fig. 2:**
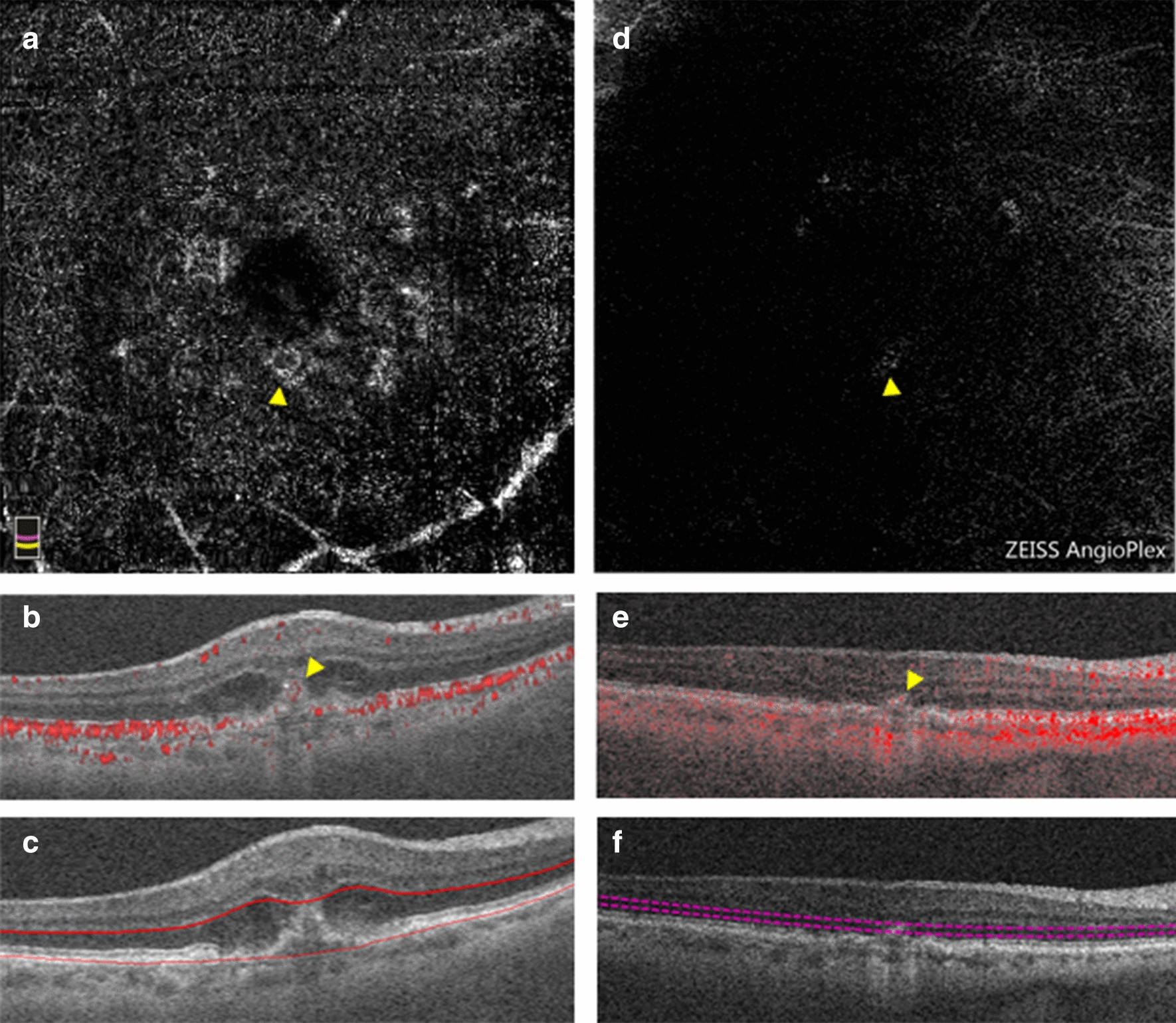
OCTA imaging of a pure type 3 MNV. **a**–**c** Baseline OCTA imaging from the RTVue XR Avanti device. **a** En face OCTA image of projection between the RPE and Bruch’s membrane segmentation lines depicting the MNV vessels (yellow arrow). **b** Corresponding B-scan through the lesion showing flow signal anterior to the RPE (yellow arrow). **c** Corresponding B-scan through the lesion showing the segmentation lines. **d**–**f** Endpoint OCTA imaging from the Cirrus HD-OCT 5000 device 21 months after the baseline imaging and after 11 total intravitreal injections of anti-VEGF. **d** En face OCTA image of the outer retina slab showing the MNV flow (yellow arrow). **e** Corresponding B-scan through the lesion with flow signal (yellow arrow). **f** Corresponding B-scan through the lesion showing segmentation lines for the outer retina

Twenty-six lesions were classified as purely type 1 MNVs. There was one lesion graded as purely type 2 (Fig. 1) and 11 considered to be mixed type 1 and type 2 MNVs, with a total of 12 MNVs considered to have a type 2 component. Five total lesions were graded to have a type 3 component, with two representing pure type 3 neovascularization (Fig. 2) and three representing mixed type 1 and type 3 lesions. The demographics of patients with each MNV type are shown in Table 1. Patients whose lesions contained a type 2 component exhibited significantly worse BCVA at both the baseline and endpoint.

The growth characteristics per MNV type are represented in Table 2. The average baseline and endpoint areas of neovascular lesions with a type 3 component were significantly smaller than the average baseline and endpoint areas of type 1 and 2 MNVs (baseline p = 0.019 and endpoint p = 0.04). The endpoint vessel density was significantly lower in the type 1 lesions compared to the other types (p = 0.05), although there was no significant difference in vessel density at the baseline across lesion types. There was no difference between the baseline and endpoint MNV area (p = 0.785 for type 1, p = 0.557 for type 2, and p = 0.75 for type 3) or vessel density (p = 0.627 for type 1, p = 0.492 for type 2, and p = 1 for type 3) within each MNV type.

**Table 2:** Growth characteristics per MNV type

	Type 1	Type 2	Type 3	*p*
MNV area, mm^2^				
Baseline	3.4 ± 3.26	2.84 ± 2.28	0.34 ± 0.53	0.019*
Endpoint	3.28 ± 3.46	2.96 ± 1.98	0.35 ± 0.28	0.04*
MNV vessel density, mm^2^/mm^2^				
Baseline	0.35 ± 0.21	0.53 ± 0.72	0.35 ± 0.28	0.715
Endpoint	0.37 ± 0.18	0.61 ± 0.33	0.59 ± 0.2	0.05*
Growth rate, %/year (n)	7.17 ± 53.03 (21)	28.51 ± 50.27 (10)	− 62.923 ± 19.97 (3)	0.01*
% shrink (n)	42.86% (9)	30% (3)	100% (3)	0.099
% stable (n)	28.57% (6)	20% (2)	–	0.525
% grow (n)	28.57% (6)	50% (5)	–	0.224

*p ≤ 0.05

**Fig. 3:**
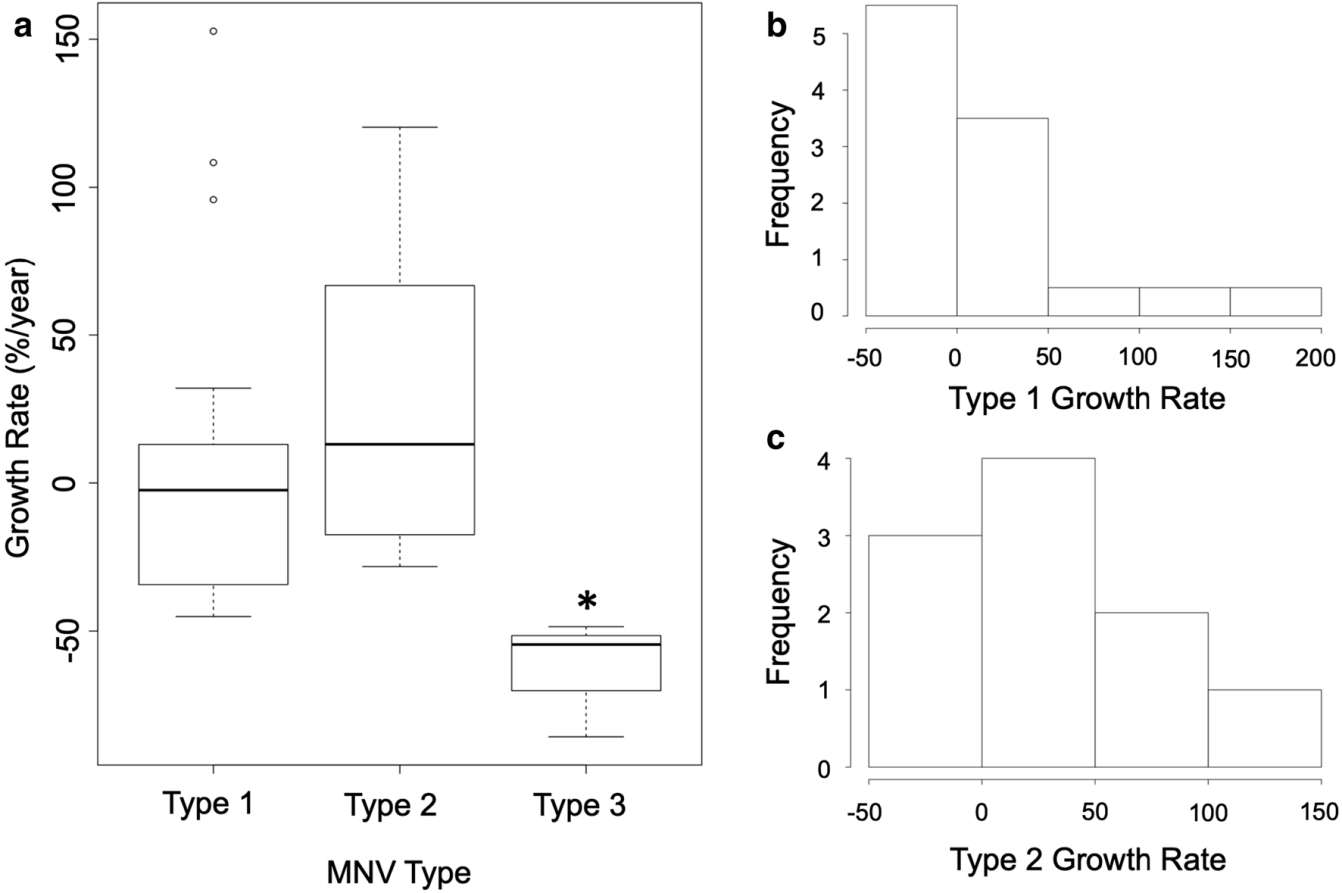
Comparing growth rates across MNV type. **a** Boxplot showing the growth rate of MNV lesions per type. *p ≤ 0.05. **b** Histogram of type 1 MNV growth rate frequency. **c** Histogram of type 2 MNV growth rate frequency

**Fig. 4:**
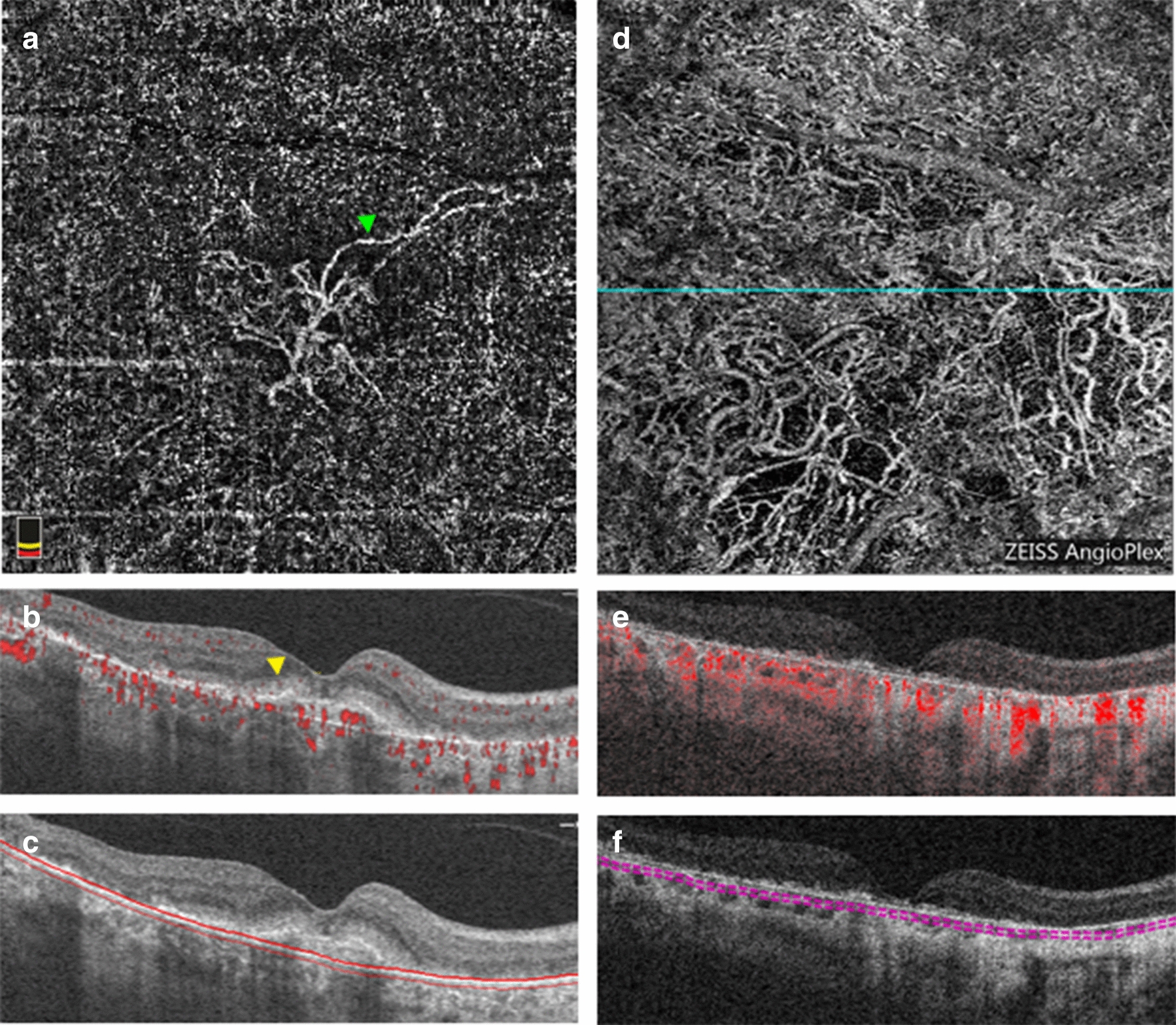
OCTA imaging of a mixed type 1, type 2 MNV before treatment and after 25 months of follow-up that regressed with 5 injections of anti-VEGF. **a**–**c** Baseline OCTA imaging from the RTVue XR Avanti device. **a** En face image from the choriocapillaris revealing the MNV network. Green arrow demarcates an example of a mature vessel. **b** Corresponding B-scan through the lesion revealing sub-RPE and subretinal (yellow arrow) flow. **c** Corresponding B-scan through the lesion showing choriocapillaris segmentation lines and hypertransmission in areas of atrophy. **d**–**f** Endpoint OCTA imaging from the Cirrus HD-OCT 5000 device. **d** En face image from the choriocapillaris without clear MNV boundaries identified but prominent choroidal vasculature visible. Cyan line denotes the cross-sectional location through which the B-scan was derived. **e** Corresponding B-scan through the center of the image with flow signal showing RPE loss, hypertransmission due to atrophy that now extends to the foveal center, and no aberrant flow signal attributable to MNV. **f** Corresponding B-scan through the center of the image showing choriocapillaris segmentation lines

**Fig. 5:**
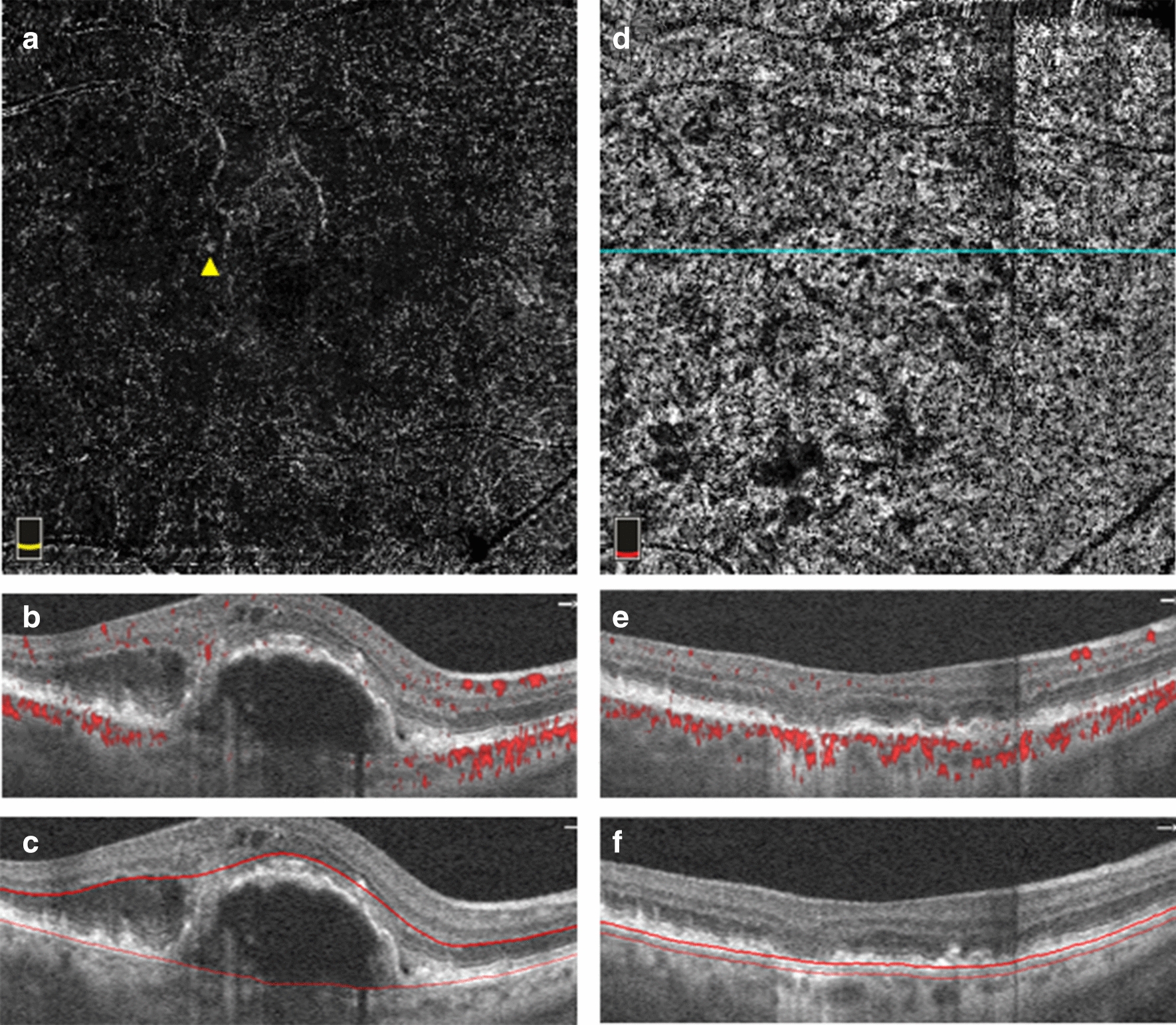
OCTA imaging of a mixed type 1, type 3 MNV before treatment that regressed within 18 months of follow up with 4 injections of anti-VEGF. **a**–**c** baseline OCTA imaging from the RTVue XR Avanti device. **a** En face image of the outer retinal slab showing flow that corresponds to the type 3 MNV component of the MNV (yellow arrow). **b** Corresponding B-scan through the lesion with flow signal. **c** Corresponding B-scan through the lesion showing outer retinal slab segmentation lines. **d**–**f** Endpoint OCTA imaging from the RTVue XR Avanti device. **d** En face image of the choriocapillaris after 18 months of follow-up. Cyan line denotes the cross-sectional location through which the B-scan was derived. **e** Corresponding B-scan through the region of atrophy revealing no intraretinal or sub-RPE flow signal. **f** Corresponding B-scan through the region of atrophy showing the choriocapillaris segmentation lines

**Fig. 6:**
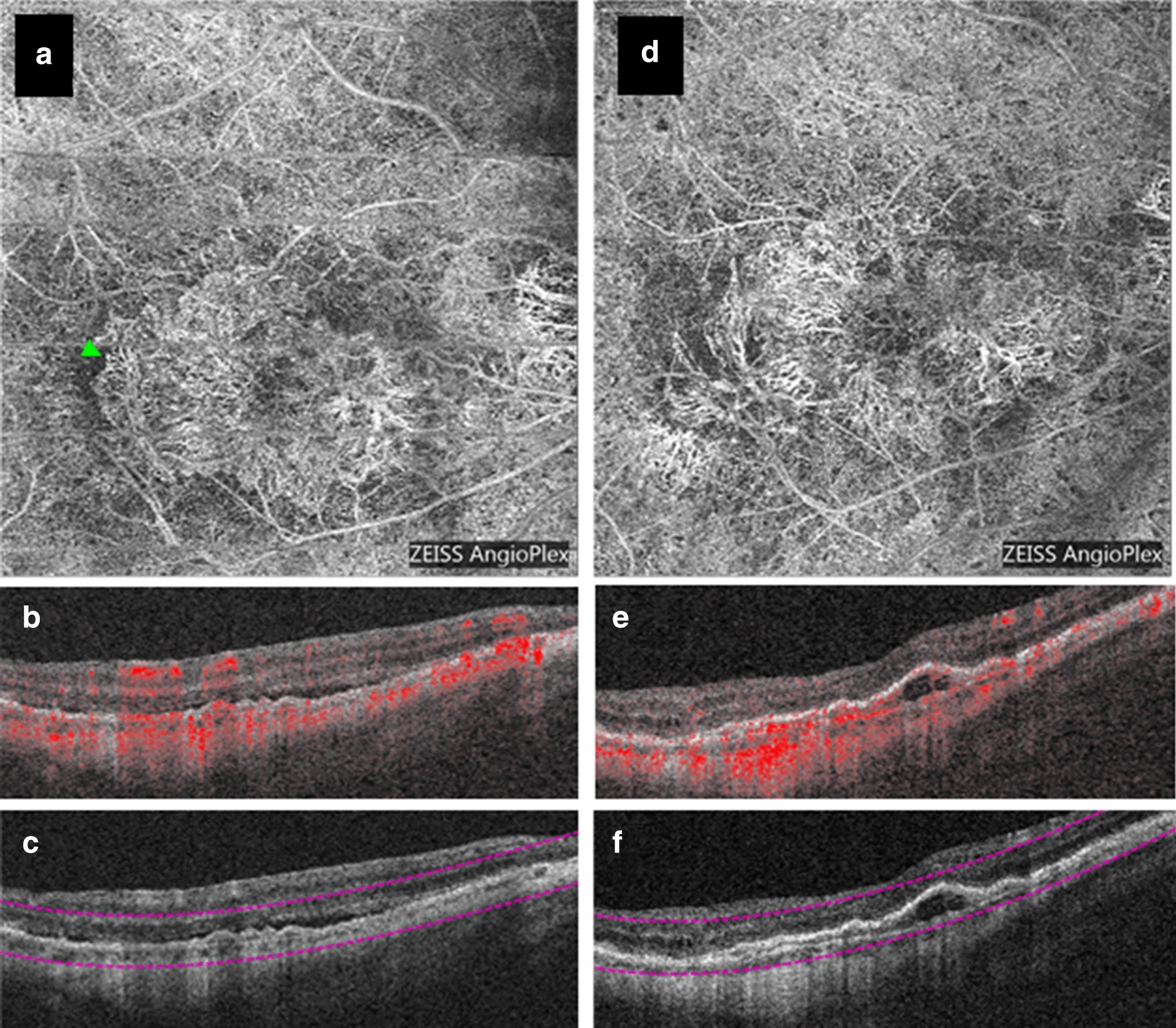
OCTA imaging of a type 1 MNV with 6 total injections of anti-VEGF that exhibited growth over a 20-month follow-up period. **a**–**c** Baseline OCTA images from the Cirrus HD-OCT 5000 device. **a** En face image from the outer retina-choriocapillaris (ORCC) slab revealing the MNV network. Green arrow demarcates an example of immature vessels. **b** Corresponding B-scan through the lesion with flow signal revealing a double layer sign. **c** Corresponding B-scan through the lesion showing the ORCC segmentation lines. **d**–**f** Endpoint OCTA imaging from the Cirrus HD-OCT 5000 device. **d** En face image from the ORCC slab revealing the MNV network. **e** Corresponding B-scan through the lesion showing with flow signal and an enlarged pigment epithelial detachment. **f** Corresponding B-scan through the lesion showing the ORCC segmentation lines

The growth rate was significantly different between type 3 MNVs when compared to type 1 and type 2 lesions (p = 0.01) (Fig. 3a). Two out of 45 lesions were considered to have completely regressed by their respective endpoint dates (Figs. 4, 5). These will be discussed further as individual cases in the following section. Figure 6 depicts an example of a type 1 MNV that grew over the follow-up period for comparison.

In an attempt to further quantify the growth patterns of lesion types, the percent of lesions per type that grew, remained stable, or shrunk over time was calculated. It is important to note that not all MNV areas could be accurately measured *en face* for both baseline and endpoint images. For this reason, growth rate and growth category were only assessed for lesions that could be traced at both baseline and endpoint visits. The number of lesions used for growth rate and category calculations are noted in Table 2. For example, Table 2 shows the baseline and endpoint MNV areas for type 3 lesions to be 0.34 ± 0.53 and 0.35 ± 0.28, respectively. However, when only cases with both baseline and endpoint areas recorded were considered (n = 3), the average type 3 MNV area was 0.12 ± 0.07 at baseline and 0.01 ± 0.01 at the endpoint. Out of the three categories (grow, stable, shrink), all type 3 MNVs (100%) shrunk and most type 1 MNVs (43%) shrunk over time, while most type 2 MNVs (50%) grew (Table 2). Chi-square analysis of growth category frequency per type revealed no significant difference in these percentages (p = 0.099 for “shrink”, p = 0.525 for “stable”, and p = 0.224 for “grow”). The frequencies of growth rates of type 1 and type 2 MNVs are also visualized in Fig. 3b, c.

Results for Spearman correlation between various growth parameters are shown in Table 3. The baseline vessel density was significantly correlated with growth rate in the type 3 lesions (p < 0.001) but not in the type 1 or type 2 lesions. There was no correlation between the number of injections per year for any MNV type and growth rate, endpoint MNV area, or endpoint activity status. On logistic regression analysis, there was no significant association between the development of atrophy and the number of injections, baseline MNV area, baseline vessel density, or lesion growth rate (Table 4). Logistic regression results were unchanged when controlling for the number of injections on multivariate analysis. All trends noted above were unchanged when the single pure type 2 MNV was excluded from analysis in an attempt to compare type 1 MNV patterns with mixed type 1, type 2 MNV patterns.

**Table 3:** Spearman correlation results between various growth parameters

	Type 1		Type 2		Type 3	
	rho	*p*	rho	*p*	rho	*p*
Growth rate vs.						
Baseline VD	− 0.209	0.367	− 0.137	0.676	1	< 0.001***
Number of injections/year vs.						
Growth rate	− 0.017	0.555	0.128	0.934	0.5	0.667
Endpoint MNV area	0.151	0.775	− 0.296	0.386	− 0.4	0.6
Endpoint activity status	− 0.067	0.761	− 0.318	0.313	− 0.775	0.225

***p ≤ 0.001

**Table 4:** Univariate logistic regression analysis comparing development of atrophy at the endpoint and MNV parameters

Parameter	OR	Estimates	SE	*p*
Number of injections	0.99	− 0.007	0.048	0.880
Baseline MNV area	0.82	− 0.204	0.23	0.376
Baseline VD	0.83	− 0.185	1.042	0.859
Growth rate	1	0.0003	0.008	0.966

Cases in which atrophy was present at the baseline were excluded from this analysis (n = 34 used in analysis)

Lastly, anti-VEGF agent analysis showed no difference in average growth rate (p = 0.967), endpoint MNV area (p = 0.3438), or endpoint activity status (p = 0.1106) across agent categories.

### Cases of complete regression

#### Case 1

A 66-year-old Caucasian woman with a history of anti-VEGF injections for nAMD and macular atrophy in the left eye presented with worsening vision in the right eye. Her visual acuity was 20/70 in the right eye, down from a baseline of 20/30. Her visual acuity in the left eye was count fingers. She was found to have a submacular mixed type 1, type 2 MNV with overlying fluid in the right eye on OCTA (Fig. 4a–c) and diffuse macular atrophy on color fundus photography and fundus autofluorescence. She had resolution of fluid one month after an intravitreal injection of aflibercept, and then was followed for 13 months with PRN treatment and received a total of 5 aflibercept injections. Her most recent OCTA at 25 months after initiation of therapy showed complete absence of flow with expansion of atrophy towards the fovea (Fig. 4d–f). Her BCVA in the right eye was 20/300 at the endpoint.

#### Case 2

A 74-year-old Caucasian woman with a history of hypertension presented to retina clinic for worsening vision. Her visual acuity was 20/50 in the right eye (baseline vision and vision in the left eye unknown). She was found to have a chorioretinal anastomosis, consistent with a mixed type 1 and type 3 MNV in the right eye on OCTA (Fig. 5a–c). She received 3 monthly loading doses of ranibizumab, with flattening of the pigment epithelial detachment and resolution of subretinal fluid after the first dose. She was then followed with PRN treatment and required only one additional injection after a three-month interval. Her last injection of ranibizumab was given 6 months after her initial presentation and 9 months prior to the last OCTA image on file (Fig. 5d–f). Her BCVA in the right eye was 20/30 at the endpoint.

## Discussion

The complete regression of MNV lesions in various retinal disease states is well known among clinicians, but to our knowledge, this is the first study to systematically assess the regression and growth response of the different MNV types in nAMD to anti-VEGF treatment. Though sample size limits our conclusions, evidence from this study suggests that full MNV regression is rare. Only two cases regressed in this cohort, and both were mixed-type lesions (Cases 1 and 2).

We postulate that low MNV regression rates in response to anti-VEGF treatment are due, in part, to the effects of extracellular matrix composition and cell–cell interactions involved in angiogenic signaling mechanisms [30]. Within the outer retina, choroidal endothelial cells (CECs) that are activated migrate and interact with Bruch’s membrane and the RPE. This environment contrasts from conditions of retinal neovascularization, such as PDR, in which endothelial cells interact with the inner limiting membrane (ILM) and vitreous. Although both forms of neovascularization may share common factors and mechanisms of angiogenesis, such as hypoxia-induced factor-1 (HIF-1) and VEGF, clinical studies report differences in outcomes following anti-VEGF therapy [31]. For example, large controlled studies looking at PDR regression in response to anti-VEGF treatment have shown regression rates of up to 64% [32].

These observations may be due to differences in crosstalk and activation of signaling mechanisms involved in the two different angiogenic processes. Microscopy in epiretinal membranes associated with PDR shows narrow vessels surrounded solely by fibroblasts and occasional macrophages. In contrast, vessels in the subretinal membranes of nAMD patients are surrounded by a rich cellular environment abundant in proteoglycans and cellular components such as pericytes, fibroblasts, RPE cells and numerous macrophages and leukocytes [33].

The finding of immune cells around MNV is consistent with work identifying inflammation and choroidal endothelial cell (CEC) activation in type 1 and type 2 MNV formation [34]. According to these studies, aging and genetic predisposition incite changes in Bruch’s membrane and the RPE, such as drusen formation and deposition of oxidized lipoproteins [35]. Debris accumulation triggers inflammatory cytokine and VEGF release from RPE cells [36]. Increased VEGF levels lead to CEC activation and migration towards the RPE, where CECs proliferate to form MNVs [30, 34]. Together, inflammatory signaling and CEC activation drive MNV formation.

Previous theories have proposed that mature MNV vessels, surrounded by pericytes that supply local VEGF, are preferentially protected from anti-VEGF treatment when compared to their immature counterparts [27]. It is possible that CECs, inflammatory cells and integrins aid pericytes in MNV vascular maintenance. As circulating VEGF levels drop with anti-VEGF treatment, the vascular endothelium and its supporting cells may become more dependent on these local survival signals for sustenance [37]. This may be a plausible theory as to why MNV vasculature, supported by a rich extracellular milieu, is less prone to regression with anti-VEGF treatment.

Despite similarities in the developmental pathway of type 1 and type 2 MNVs, the two types cannot be uniformly grouped together [38]. Recent studies investigating the behavior of type 1 and type 2 MNVs exposed to anti-VEGF treatment have shown conflicting results. Kim et al. compared the responses of both type 1 and type 2 MNVs to anti-VEGF treatment [39]. This group found no significant change in size between baseline and 12 months of follow up in type 1 MNVs, but found a significant decrease in type 2 MNV area for the same follow-up window [39]. McClintic et al. found a greater reduction in the area of type 2 MNV compared to the area of type 1 MNV after 1 month of anti-VEGF treatment, but the reduction in area was no longer statistically significant at the 1 year follow up for either MNV type [40]. On the contrary, Xu et al. evaluated the long-term evolution of only type 1 MNVs exposed to anti-VEGF treatment for a 12–27 month time period, and found 80% of lesions exhibited a slight increase in area [41].

The current study saw more type 2 MNVs grow during the follow-up period compared to type 1 MNVs (50% versus 28.57%), however this result did not reach statistical significance. Overall, the frequencies of growth categories across MNV type are not statistically significant. Similarly, there was no significant difference between the baseline and endpoint MNV areas within a given MNV type nor was there a significant difference in the magnitude of growth rate across types. This means that even though lesions generally shrunk or grew, there was no significant change in size from the baseline measure overall. Trends noted in this study suggest that the presence of a type 2 component might lend to the risk for expansion, though future, larger studies are required to evaluate this idea.

Finally, this study did not find a correlation between the number of anti-VEGF injections and the final MNV area or the growth rate in type 1 or type 2 MNVs, a phenomenon that was also observed by Xu et al. [41]. There was also no relationship between anti-VEGF agent or combination of agents and growth rate, or endpoint area or activity. Taken together, these results suggest that anti-VEGF exposure is not correlated with the vascular growth rate of a choroidal MNV, and that agents perform similarly with respect to morphological outcomes. The observations support the view that anti-VEGF therapy can control the activation of CECs, thus preventing further growth. Of note, this study did not control for anti-VEGF administration regimen. Future controlled studies are required to pinpoint the relationship, if any, of anti-VEGF injections to incremental growth trends over a longer follow-up duration.

In the present study, the few cases of type 3 MNVs that were included shrunk in the follow-up period with anti-VEGF treatment. Several groups using OCTA have reported complete regression of type 3 MNVs after anti-VEGF treatment in 29–48.7% of cases [23, 24, 42]. The most recent comprehensive study on type 3 MNVs using OCTA, however, suggests these lesions do not completely regress with treatment and, instead, can recur [42]. The origins of type 3 MNVs have been highly debated. A recently published model proposes a retinal origin for type 3 lesions driven by VEGF expressed from the underlying hypoxic RPE or RPE cells that had migrated within the retina. This model suggests that new vessels grow in response to high VEGF levels and extend towards the outer retina following a VEGF gradient and widening in vascular caliber to accommodate increasing flow demand [5, 43]. Other studies have suggested Müller cell expression of VEGF [3]. Future studies assessing the retinal vasculature prior to type 3 MNV development could elucidate the origin of these lesions.

Along with assessing the regression and growth rates across MNV types, this study also sought to correlate quantitative OCTA metrics, such as vessel density, with growth rate. There was no correlation between baseline vessel density and growth rate in the type 1 and type 2 lesions. This is consistent with the report by Kim et al. that who found no significant changes in vessel density for both type 1 and type 2 MNVs after treatment and no correlation between vessel density and the rate of change of MNV size [39]. Xu et al. also found no correlation between vessel density and growth rate in type 1 MNVs [41]. In the current study, the endpoint vessel density of type 1 MNVs was significantly smaller than the endpoint vessel density of type 2 and 3 MNVs. However, there was no significant difference between vessel densities across MNV types at baseline. These results suggest the limited role of this metric as a proxy for MNV maturation or growth.

This study also attempted to investigate the effects of anti-VEGF exposure on vessel maturity. The process of maturation with anti-VEGF exposure is attributed to cycles of low VEGF levels leading to the regression of new vascular sprouts and the resulting high flow stimulus for vessel dilation, or arteriogenesis, which is not VEGF dependent [27]. Though conceptually interesting, it has proven difficult to accurately classify vessels into mature versus immature types using OCTA. An initiative of experts in the field found that there was poor consensus in distinguishing immature versus mature vessels across graders (unpublished data from the Unified Commentary of the Committee of International Experts on the Nomenclature for Neovascular AMD in OCTA). The present study also attempted to codify immature versus mature vessels. Distinguishing these vessel types was particularly challenging in type 3 MNVs where the lesions appear as small, bright tufts without clearly defined vessels on OCTA [17, 44, 45]. Due to the lack of agreement among graders in this study, we were unable to draw conclusions from the classification of vessels as mature or immature. The lack of overall consensus for this classification suggests that the determination of vessel maturity is not a sufficiently reproducible biomarker for investigative studies.

Finally, the association with the development of atrophy and anti-VEGF use has been hotly debated in recent years, but this study found no significant association between the development of atrophy and anti-VEGF exposure or MNV growth variables [46]. Though a clear correlation with the development of atrophy was not expected, it is possible that macular atrophy was under identified since, for some subjects, analysis was restricted to 3 × 3 mm images, the length of follow-up may not have been long enough to detect this effect, and the type and amount of anti-VEGF treatment may be different than other studies that have suggested this association.

The two cases of regression posit an interesting discussion about the relationship between atrophy and MNVs. In Case 1, the endpoint image revealed a large area of atrophy (Fig. 4d–f), and the abnormal neovascular complex was classified as completely regressed because there were no abnormal findings on B-scan, although the vessels could still be visualized intrachoroidally (Fig. 4d). We hypothesize the large area of atrophy may have contributed to the inward-shrinking of these vessels. The RPE acts as a source of VEGF for MNV lesions, and atrophy disrupts the RPE, therefore reducing underlying VEGF levels necessary for MNV survival [47]. The regression of the lesion in Case 2 was also accompanied by the development of atrophy. However, we are severely limited by number to make any thorough assumptions, and the relationship between atrophy and MNV regression warrants further longitudinal investigation.

There are several limitations to this investigation. First and foremost, the retrospective nature of this study lends itself to faults. As mentioned previously, the small sample size, which was dictated by the inclusion criteria of the retrospective screen, is not large enough to obtain robust data on expansion or regression rates for the different types of MNV lesions. Furthermore, the small number of qualifying cases meant that only a few cases of pure type 2 and type 3 lesions were studied. This necessitated the combination of mixed lesions with a type 1 component into the aforementioned categories, which could confound findings. Despite possible confounding, the number of mixed-type lesions found in this screen suggests these lesions are common and should not be excluded from further study simply due to their varied components. In fact, type 2 MNVs rarely exist in a pure state. Choroidal vasculature must break through the RPE and proliferate under the retina to form these lesions. This suggests that type 2 lesions likely proliferate from underlying type 1 lesions. It is, therefore, unclear if a type 2 lesion can truly be pure at all, or if the type 1 component is simply not visible in seemingly pure cases. For these reasons, we felt it necessary to group lesions by components.

Treatment regimen and follow-up duration were not controlled for in this study. This can be seen as a study strength since our cohort reflects a more generalizable clinical sample. However, not controlling for treatment regimen and follow-up duration introduces variability and limits our ability to find significant differences across MNV types. Another limitation to this study is the fact that grading was not independent of examination date. Graders were required to search for specific images within the device software to assess *en face* images with their corresponding B-scans. This method increases the risk of biased evaluation since all images are listed by acquisition date on device platforms. Finally, patients were imaged on a variety of OCTA devices, which further contributes to variability. MNV area, while reproducible across different spectral-domain OCTA devices, is variable between spectral domain and swept-source OCTA technology [48, 49]. Furthermore, other MNV quantitative parameters, such as vessel density, are not reproducible across OCTA devices [50]. Larger prospective studies that control for imaging device and treatment regimen could provide more granular insights into the growth and regression rates across MNV types in nAMD.

## Conclusions

Anti-VEGF treatment is not associated with vascular regression on OCTA in nAMD. This work emphasizes the clinical role of anti-VEGF as an anti-leakage agent rather than a vascular regression agent. This study also suggests that the regression pattern of neovascularization that originates in the choroid is different to that which originates in the retinal vasculature. Future studies are required to determine if OCTA metrics can predict how an individual MNV will behave with anti-VEGF exposure.

## Data Availability

The datasets used and/or analyzed during the current study are available from the corresponding author upon reasonable request.

## Abbreviations

AMD: Age-related macular degeneration
Anti-VEGF: Anti-vascular endothelial growth factor
BCVA: Best corrected visual acuity
CEC: Choroidal endothelial cells
cRORA: Complete RPE and outer retinal atrophy
FA: Fluorescein angiography
HIF-1: Hypoxia induced factor-1
ILM: Inner limiting membrane
iRORA: Incomplete RPE and outer retinal atrophy
MNV: Macular neovascularization
nAMD: Neovascular age-related macular degeneration
OAT: OCTA analysis toolbox
OCT: Optical coherence tomography
OCTA: Optical coherence tomography angiography
PDR: Proliferative diabetic retinopathy
PRN: Pro re nata
RPE: Retinal pigment epithelium
VEGF: Vascular endothelial growth factor
